# The Role of Brain-Derived Neurotrophic Factor and Glial Cell Line-Derived Neurotrophic Factor in Chronic Fetal Oxygen Deprivation

**DOI:** 10.17691/stm2020.12.1.03

**Published:** 2020

**Authors:** N.A. Shchelchkova, A.A. Kokaya, V.F. Bezhenar’, O.V. Rozhdestvenskaya, M.A. Mamedova, T.A. Mishchenko, E.V. Mitroshina, M.V. Vedunova

**Affiliations:** Associate Professor, Department of Neurotechnologies, Institute of Biology and Biomedicine, National Research Lobachevsky State University of Nizhni Novgorod, 23 Prospekt Gagarina, Nizhny Novgorod, 603950, Russia, Head of Molecular and Cellular Technologies Department, Institute of Fundamental Medicine, Privolzhsky Research Medical University, 10/1 Minin and Pozharsky Square, Nizhny Novgorod, 603005, Russia; Obstetrician-Gynecologist, Pavlov University, 6–8 L’va Tolstogo St., Saint Petersburg, 197022, Russia; Professor, Head of the Department of Obstetrics, Gynecology and Reproductology, Pavlov University, 6–8 L’va Tolstogo St., Saint Petersburg, 197022, Russia; Senior Laboratory Assistant, Department of Obstetrics, Gynecology and Reproductology, Pavlov University, 6–8 L’va Tolstogo St., Saint Petersburg, 197022, Russia; Assistant, Department of Obstetrics, Gynecology and Reproductology, Pavlov University, 6–8 L’va Tolstogo St., Saint Petersburg, 197022, Russia; Senior Researcher, Laboratory for Neuroprotection Methods Development, Center for Translational Technologies, National Research Lobachevsky State University of Nizhni Novgorod, 23 Prospekt Gagarina, Nizhny Novgorod, 603950, Russia, Senior Researcher, Molecular and Cellular Technologies Department, Institute of Fundamental Medicine, Privolzhsky Research Medical University, 10/1 Minin and Pozharsky Square, Nizhny Novgorod, 603005, Russia; Associate Professor, Department of Neurotechnologies, Institute of Biology and Biomedicine, National Research Lobachevsky State University of Nizhni Novgorod, 23 Prospekt Gagarina, Nizhny Novgorod, 603950, Russia, Senior Researcher, Laboratory for Neuroprotection Methods Development, Center for Translational Technologies, National Research Lobachevsky State University of Nizhni Novgorod, 23 Prospekt Gagarina, Nizhny Novgorod, 603950, Russia, Senior Researcher, Molecular and Cellular Technologies Department, Institute of Fundamental Medicine, Privolzhsky Research Medical University, 10/1 Minin and Pozharsky Square, Nizhny Novgorod, 603005, Russia; Leading Researcher, Institute of Biology and Biomedicine, National Research Lobachevsky State University of Nizhni Novgorod, 23 Prospekt Gagarina, Nizhny Novgorod, 603950, Russia, Director of Institute of Biology and Biomedicine, National Research Lobachevsky State University of Nizhni Novgorod, 23 Prospekt Gagarina, Nizhny Novgorod, 603950, Russia

**Keywords:** brain-derived neurotrophic factor, BDNF, glial cell line-derived neurotrophic factor, GDNF; neuron-specific enolase, NSE, hypoxia-inducible factor, HIF-1β, prenatal hypoxia

## Abstract

**The aim of the study** was to define the role of brain-derived and glial cell line-derived neurotrophic factors (BDNF and GDNF) in realization of compensative and adaptive mechanisms of a neonatal organism to hypoxia.

**Materials and Methods:**

The experiments *in vivo* have been carried out on pregnant C57BL/6 mice (n=36). Chronic hypobaric hypoxia has been modeled in different pregnancy trimesters. On gestation days E19–20, concentration of BDNF and GDNF in the blood of the pregnant females was determined by enzyme immunoassay. Further, the number of neonatal mice, their weight and body length parameters have been assessed.

Parturient mothers (n=88) and their newborn babies followed up at the Clinic of Obstetrics and Gynecology of Pavlov University took part in the clinical investigations. Concentration of BDNF, GDNF, neuron-specific enolase (NSE), and hypoxia-inducible factor (HIF-1β) in the fetal cord blood has been determined by ELISA. The obtained data were retrospectively compared with cardiotocography, dopplerometry, presence of meconium-stained amniotic fluid and the neonate state at birth, assessment according to the Apgar score, and the course of adaptation period.

**Results:**

Chronic hypobaric hypoxia in pregnant mice in trimester I and II resulted in the significant decrease of BDNF and GDNF level, decrease in the number of embryos, and in significant changes in weight/height characteristics of the newborn pups.

According to the clinical observations, an increased expression of the neurotrophic factors BDNF, GDNF provides protection to a neonate even if hypoxia factors are present and realized. A low content of BDNF and GDNF was observed in the group of infants with a high risk of developing unfavorable hypoxic damaging effects.

**Conclusion:**

The protective role of BDNF and GDNF in the regulation of fetal homeostasis in chronic hypoxia has been established experimentally and clinically.

## Introduction

Prenatal hypoxia occupies a special place among hypoxic states of various genesis. The problem of assessing the compensatory and adaptive mechanisms in a fetus during pathological pregnancy and at labor formed as early as 50s years of the last century [1] and has been of current importance to present days. Complications associated with perinatal fetal hypoxia develop in 10% of the total number of deliveries. The diagnosis and especially treatment of hypoxia in obstetrics and neonatology are connected with substantial difficulties that can be explained by insufficient understanding of molecular mechanisms of hypoxic damage.

In recent years, keen interest of researchers is aimed at studying the diagnostic and prognostic value of determining the concentration of neurotrophic factors in the body tissues and fluids. These regulatory proteins participate actively in the formation and development of neuronal outgrowths, development of cerebral cortex, and in the processes of synaptic plasticity [2, 3]. Neurotrophic factors play a crucial role in neuroprotection, promote the survival of nerve cells, prevent the development of apoptotic reactions in many neuronal populations, and, consequently, can influence pre- and postnatal brain development [4, 5].

One of the main functions of the brain-derived neurotrophic factor (BDNF) is to maintain the viability of neurons not included in the neural network structure in the early postnatal period. Mature neurons, which do not have sufficient number of connections with other neurons, are characterized by an activation of apoptosis and subsequent death. BDNF is able to support the structural and functional arrangement up to the completion of neuronal network formation under the influence of afferent stimuli in the postnatal period [6–8].

Glial cell line-derived neurotrophic factor (GDNF) is known as an effective neuroprotector in the development of various pathologies, including ischemia, capable to preserve both the viability of nerve cells and functional metabolic activity of neuronal networks and the structure of the synaptic apparatus under stress conditions [9, 10].

As the neurotrophic factors, apart from the local effects, play an important role in the interaction of the nervous and visceral systems and cause a systemic effect on the organism, they are able to pass through the blood-brain barrier and be detected in the blood flow at any age. Determining the content of the neurotrophic factors and other proteins of the nervous system in blood serum can substantially extend the diagnostic potential in the assessment of CNS.

Quantitative determination of neuron-specific enolase (NSE) level is a promising marker of the degree of neuronal damage. This specific neuronal enzyme normally has intracellular localization and is detected in the intercellular space and blood flow only in case of cellular membrane destruction and neuronal death. The NSE concentration in this case correlates with the number of dead cells [11, 12].

In recent times, research into the mechanisms of hypoxic damage has attached particular importance to the study of HIF family proteins. These proteins govern the resistance of cells, including neurons, to oxygen deprivation conditions and are considered as key participants of molecular triggering mechanisms of the cell tolerance to hypoxia [13].

Since clinical trials have many limitations, the study of pathological mechanisms triggered in chronic intrauterine hypoxia is carried out using special experimental animal models. Fundamental studies provide the possibility to form a more distinct concept on the nature and the extent of the CNS damage in hypoxic states enabling the researchers to develop a new strategy of therapeutic correction of this pathology in near future [14–16]. In this regard, we assume that detection of BDNF and GDNF concentrations in tissues and biological liquids may allow estimating adaptive and compensatory capabilities of a newborn that has undergone hypoxia.

**The aim of the study** was to define the role of BDNF and GDNF neurotrophic factors in realization of compensative and adaptive mechanisms of a neonatal organism to hypoxia.

## Materials and Methods

***In vivo studies.*** The experiments have been carried out on pregnant C57BL/6 mice in different gestation periods (n=36). The animals were kept in a certified vivarium of the National Research Lobachevsky State University of Nizhni Novgorod in compliance with Order No.1179 of USSR MH of October 11, 1983 and Order No.267 of RF MH of June 19, 2003. The work was carried out in accordance with the Guide for the Care and Use of Laboratory Animals (National Research Council, 2011) and the requirements of the European Convention for the Protection of Vertebrate Animals used for Experimental and Other Scientific Purposes (Strasbourg, 2006). The investigation was approved by the Ethical Committee of the National Research Lobachevsky State University of Nizhni Novgorod.

Chronic hypobaric (altitude) hypoxia (CHH) was used as a convenient experimental model for antenatal hypoxia investigation. Reduction of oxygen content in the altitude chamber is a physiologically adequate correctable effect that makes it possible to apply it in various modes.

In the *first series* of the experiments, CHH modeling was performed on pregnant mice in different periods of gestation: trimester I on days E1–7 (n=6), trimester II on days E7–14 (n=6), trimester III on days E14–21 (n=6). These experiments allowed us to define the period of pregnancy at which an embryo is most vulnerable to unfavorable hypoxic damaging effects. Animals were placed into the hypobaric chamber under the pressure of 405–350 mm Hg (corresponding to the altitude of 5500–6000 m above sea level) for 40 min. Pregnant females included into the control group were not exposed to CHH (n=6). On day E19, embryos were removed from the pregnant mice and counted.

In the *second series* of the experiments, CHH was modeled on the pregnant mice (n=6) in trimesters I and II (from gestation day E5 to E15). For this purpose, mice were exposed to the conditions corresponding to the altitude of 6500–7000 m for 30 min during 10 days in the hypobaric chamber. Pregnant mice not exposed to CHH were included into the control group (n=6). On gestation days E19–20 blood samples were collected to determine BDNF and GDNF concentrations by ELISA kits BDNF DY248 (R&D Systems, USA) and GDNF CSB-E07341M (Cusabio, USA). Then, a number of newborn mice, their weight and body length characteristics were calculated.

***Clinical trials.*** Parturient mothers (n=88) and their newborn babies followed up at the Clinic of Obstetrics and Gynecology of Pavlov University took part in these investigations. The neurotrophic factors in the cord blood serum were measured by ELISA kits BDNF DY248 (R&D Systems) and GDNF DY212 (R&D Systems). The content of NSE (cat. 8476, Vector-best, Russia) and HIF-1β (ARNT) (SED470Hu, Cloud-Clone, China) was determined in the same way. The obtained data were retrospectively compared with cardiotocography, dopplerometry, presence of meconium-stained amniotic fluid and the neonate state at birth, assessment according to the Apgar score, and the course of adaptation period which are significant clinical risk factors for the development of fetal hypoxia.

***Statistical data analysis.*** The results are presented as mean (M) ± standard error of the mean (m). The significance of differences between the experimental groups was determined using Statistica 10.0 software package and nonparametric Mann–Whitney U-test. Differences were considered significant at p<0.05.

## Results

In CHH model in pregnant mice in different trimesters (I, II, III) it has been shown that the most adverse effects on the number of embryos occurred when they were exposed to hypoxia during trimester I and II of intrauterine development. In these experimental groups, the number of pups was significantly less compared to the control values. No such effects were found in pregnant mice in the third trimester, the number of embryos was comparable with the control group (Table 1). Thus, the embryos whose period of development corresponds to pregnancy trimesters I and II have been established to be most vulnerable to oxygen deficiency.

**Table 1:** Average number of fetuses in the group of C57BL/6 mice on gestation days E19–20 after chronic hypobaric hypoxia modeling in different trimesters of pregnancy (M±m)

Groups	Trimester I, days E1–7 (n=6)	Trimester II, days E7–14 (n=6)	Trimester III, days E14–21 (n=6)
Control (n=6)	10.9±0.8	11.2±0.7	9.8±0.6
Experiment (n=18)	5.8±1.2*	6.2±1.7*	8.6±1.6

* Statistically significant differences from the control group; p<0.05, Mann–Whitney test.

After definition of the critical periods of ontogenesis in which impairment of oxygen supply resulted in the decreased quantity of embryos, the second series of the experiments was carried out to investigate the effect of CHH on the morphometric parameters of pups with a more prominent hypoxic damage. In the period of trimesters I–II (gestation days E5–15), female mice were daily “elevated” in the altitude chamber to the simulated height of 6500–7000 m for 40 min with subsequent observation of the litter for 3 days (Table 2).

**Table 2:** Morphometric parameters of newborn mice exposed to chronic hypobaric hypoxia in trimesters I–II of pregnancy (on average across the group) (M±m)

Groups	Number of fetuses	At birth		Day 3 of postnatal period	
		Weight (g)	Length (сm)	Weight (g)	Length (сm)
Control (n=6)	11.2±1.2	0.81±0.02	2.19±0.12	4.14±0.35	4.15±0.53
Experiment (n=6)	5.8±0.9*	0.17±0.04*	0.68±0.06*	2.0±0.08*	3.09±0.42*

* Statistically significant differences from the control group; p<0.05, Mann–Whitney test.

The exposure to CHH in trimesters I–II has been found to result in significant decrease of embryo quantity relative to the control group (p<0.05, Mann–Whitney test). Changes in weight/length characteristics in the delivered litter have been also analyzed. An average weight of the newborn mice exposed to CHH was 0.17±0.04 g, i.e. 4 times lower than in the control group (0.81±0.02 g). In the experimental group there was, on average, a 3-fold reduction of body length (0.68±0.06 cm) in comparison with the control (2.19±0.12 cm) (р<0.05, Mann–Whitney test).

By day 3 of the postnatal period, the weight of the young mice has gradually increased but did not exceed 2.0 g in the experimental group whereas in the control animals it was already 4.14±0.35 g. The length of the mice body exposed to CHH remained also significantly shorter than the control animals (3.09±0.42 vs. 4.15±0.53 cm).

On gestation days E19–20, the BDNF and GDNF concentration in the blood plasma of the pregnant mice was determined by ELISA (Table 3). It was shown that CHH leads to the significant reduction of both factors in the female blood. The BDNF level was equal to 0.08±0.01 pg/ml, that of the GDNF 0.05±0.02 pg/ml whereas in the control group they were 0.37±0.08 and 0.15±0.06 pg/ml, respectively.

**Table 3:** Concentrations of neurotrophic factors BDNF, GDNF in blood plasma of the pregnant C57BL/6 female mice on gestation days E19–20 after chronic hypobaric hypoxia modeling (M±m)

Groups	BDNF (pg/ml)	GDNF (pg/ml)
Control (n=6)	0.37±0.08	0.15±0.06
Experiment (n=6)	0.08±0.01*	0.05±0.02*

* Statistically significant differences from the control group; p<0.05, Mann–Whitney test.

It may be concluded that prenatal hypoxia affects not only the quantity and morphometric characteristics of the neonatal mice but causes inhibition of BDNF and GDNF synthesis that undoubtedly influence their capability to realize adaptive mechanisms in the postnatal period.

Next we analyzed the BDNF and GDNF level in the cord blood of the babies exposed to prenatal hypoxia of various genesis mainly in pregnancy trimesters I and II.

The analysis of BDNF and GDNF concentration values in the neonates’ cord blood allowed us to divide all pregnant women participated in our investigation into 2 subgroups (40 and 48 women, respectively) with a high and low level of neurotrophic factors in their newborns. The difference between the concentrations of these factors in the groups was, on average, 98% for GDNF and 40% for BDNF (Table 4).

**Table 4:** Concentrations of neurotrophic factors BDNF, GDNF in the cord blood plasma of the newborns (M±m)

Subgroups	BDNF (ng/ml)	GDNF (pg/ml)
Subgroup 1 (n=40)	9.40±1.40	4.77±0.04
Subgroup 2 (n=48)	5.67±1.20*	0.10±0.01*

* Statistically significant differences from subgroup 1; p<0.05, Mann–Whitney test.

The subsequent retrospective analysis of labor case histories with emphasis on the significant risk factors of fetal hypoxia development has shown that in the subgroup with a high content of BDNF and GDNF, hypoxic factors level were high in 69.5% of cases, doubtful in 21.7% of cases, and in 8% these factors were not revealed, but the outcome for the fetus was favorable in all the cases.

In the subgroup with low values of BDNF and GDNF, the retrospective analysis has found high hypoxic factors level in 75% of cases and doubtful in 25% of cases. The outcome for the fetuses was unfavorable in 75% of cases and required intensive therapy, transfer to the hospital and/or additional consultation with neonatologist.

Regulation of adaptation to hypoxia is closely related to different molecular and cellular processes in which one of the main roles belongs to the transcriptional complex of hypoxia-inducible factor (HIF). On the other hand, hypoxia causing activation of some pathological processes associated with cell death results in the increase of specific intracellular proteins, including NSE, in the blood flow, the level of which correlates with the degree of tissues/organs injury.

In this regard, concentrations of HIF-1β and NSE were additionally determined in the neonate cord blood for 30 of 88 randomly selected patients. The participants of this investigation were ranked according to the results and formed 3 subgroups (Table 5).

**Table 5:** Concentrations of neurotrophic factors BDNF, GDNF taking into account hypoxia markers NSE, HIF in the cord blood plasma of the newborns (M±m)

Subgroups	BDNF (ng/ml)	GDNF (pg/ml)	NSE (ng/ml)	HIF (ng/ml)
Subgroup 1 (n=10)	9.81±1.70	4.45±0.50	10.60±0.70	0.14±0.005
Subgroup 2 (n=12)	10.62±1.0	5.10±1.80	27.60±2.30*	0.35±0.06*
Subgroup 3 (n=8)	6.20±0.70*	0.01±0.001*	12.0±2.0	0.27±0.08*

* Statistically significant differences from subgroup 1; p<0.05, Mann–Whitney test.

Newborns whose experimentally detected values of BDNF, GDNF were high and HIF, NSE values low were referred to subgroup 1 with a low risk for hypoxia development (n=10). The retrospective analysis of labor case histories showed that in 70% of cases, the risk factors of hypoxia development were not revealed though in 30% of cases they were present. However, all neonates of this subgroup had a favorable outcome, no posthypoxic complications were observed.

Subgroup 2 with a moderate risk of hypoxia development (n=12) included the newborns with experimentally determined high values of BDNF, GDNF, and hypoxic factors HIF, NSE. Based on the retrospective analysis of labor histories, factors associated with hypoxia development in fetuses were detected in 75% of cases, in 25% they were not found. All neonates of subgroup 2 had a favorable delivery outcome.

Subgroup 3 with a high risk of hypoxia development (n=8) consisted of the neonates with minimal values of GDNF, reduced indices of BDNF and NSE, and a high level of HIF. In this subgroup, the risk factors of hypoxia development during pregnancy and at labor were present in 100% of cases. In 75% of cases, the outcome for the newborns was unfavorable: Apgar score was 7 and less, there were adaptation period disorders, some babies required resuscitation measures, and transfer to the children hospital.

## Discussion

The central nervous system is the most vulnerable link which defines the threshold of the entire organism tolerance to hypoxic conditions. Fetal hypoxia involves a long current stage pathological process the danger of which consists in deep violations of growth and differentiation of cellular elements of the brain and spinal cord.

Hypoxia affects the fetus through maternal organism and placenta. In case of oxygen deprivation, a molecular cascade of reactions is triggered resulting in the release of stress hormones to the maternal blood and structural and functional changes in the maternal and fetal parts of placenta. Intrauterine hypoxia leads to the abnormal formation of organs, one of the signs of which is their hypotrophy. As seen from the presented experimental data *in vivo*, the weight and body length of the newborn mice are significantly decreased in comparison with the control values reflecting the dependence of their development from oxygen supply to the mother’s organism.

An indisputable argument of CHH impact on the pregnancy course in trimesters I–II was the diminished size of the litter as well as low weight/length characteristics of the newborn mice. CHH was accompanied by a steady reduction of BDNF and GDNF expression in the blood of the pregnant mice.

The diagnosis of brain injury in neonates caused by prenatal hypoxia/ischemia is difficult in the majority of cases. Impossibility to apply the diagnostic methods and interpretation of the results tried and tested on adult patients leaves open the problem of intrauterine hypoxia: it is necessary to develop our own arsenal of markers and criteria.

One of the current directions in solving this problem is investigation of the role of neurospecific proteins in the formation of adaptive capabilities of a newborn organism. A neurotrophic signaling is critical for the pre-and postnatal brain development due to its influence on the process of neuron development and reaction to the perinatal stress [5].

The performed experiments have found the decrease of BDNF, GDNF content in the blood serum of the pregnant C57BL/6 mice relative to the control values which may designate a failure of adaptive mechanisms under CHH conditions. And GDNF, which is an excretory protein of glial cells (astrocytes), showed the maximal reduction of its concentration under these conditions. It should be noted that recently great importance has been attached to the pathology of glial cells when degenerative processes in human CNS take place, including ischemic/hypoxic genesis [17, 18].

The obtained results defined the appropriateness of comparing the effects of experimental and clinical hypoxia and the assessment of the neurospecific proteins as predictors of hypoxic brain injury in neonates.

It is important to mention that changes on the curve of cardiotocography or ECG and meconium-stained amniotic fluid, the clinical signs of hypoxia, do not always determine the state of a newborn, whereas presence of neurospecific proteins in fetal blood, as shown by our investigation, may serve as an indicator of “true” hypoxia.

Involvement of integral protein markers NSE and HIF as participants of pathological hypoxic processes at the molecular level enabled us to collate more distinctly concentration dependence of neurotrophic factors with realization of adaptive processes in neonates.

The retrospective analysis of labor case histories with special attention to the significant risk factors of fetal hypoxia development has shown that tolerance of a neonate organism to chronic perinatal hypoxia depends mainly on a genetically determined level of BDNF and GDNF.

This approach allowed us to come to the conclusion that relatively high concentrations of BDNF and GDNF in a combination with low values of hypoxic markers NSE and HIF form a group of newborns with a low risk of hypoxic sequelae even if hypoxic factors were present during pregnancy.

The second group of newborns was characterized by high values of hypoxia-induced proteins NSE and HIF in cord blood. Notable that NSE concentration did not exceed 40 μg/L, values which in newborns, according to the literature data, designate presence of cerebral ischemia [19]. It should be assumed that increase of gene expression of the damaging factors was inhibited by the increased synthesis of BDNF, GDNF. In the previous studies it has been shown that BDNF is able to penetrate across the blood-brain barrier [20] and its level in the neonates’ blood serum correlates with that in the brain cortex [21]. Substantial release of neurotrophic factors to the infants’ peripheral blood flow is likely to occur due to the activation of expression of these proteins and immaturity of the blood-brain barrier function. However, there are data that BDNF can be transferred from mother to fetus via blood, amniotic fluid, and placenta where the neurotrophic factors participate in the processes of placental angiogenesis and maturation, and determine also the pregnancy outcome [22–24].

The newborns with the established high concentrations of NSE, HIF, BDNF, and GDNF were united into the group of moderate risk and had a high probability of hypoxic effects. However the delivery outcome for this group was favorable which was most likely to be due to the activation of compensatory mechanisms including the regulatory system of the neurotrophic factors.

The third group of newborns was characterized by a relatively high level of the hypoxic marker HIF but low parameters of NSE and BDNF, GDNF. In such newborns, molecular pathological mechanisms of hypoxia are triggered, but realization of neuroprotective cascades involving neurotrophic factors does not occur. Therefore, these patients should be referred to a high risk group in which the development of hypoxia-induced damages is registered in 100% of cases and in 75% of cases the outcome for a fetus is unfavorable.

Our findings are in agreement with the previously published data according to which a low level of BDNF in the children cord blood should be considered as unfavorable for the periods of peculiar sensitivity of the brain to the action of the hormones and peptide regulators during pregnancy and labor. Data on GDNF content in the newborn cord blood are different depending on the gestational age of the fetus and also on the pregnancy and labor course [25]. GDNF concentration may be decreased in response to the increase of cytokine or chemokine levels in the brain parenchyma [26].

Thus, further experimental and clinical studies are necessary in order to clarify neurotrophic factors role in the prenatal period and, especially, with regard to factors that may cause neurological disorders in the developing brain and predispose to neuro- and psychopathology in later life.

## Conclusion

Chronic hypobaric hypoxia in pregnant mice in trimester I and II resulted in the significant decrease of BDNF and GDNF level, the embryo quantity, and the significant changes in weight/length parameters in the newborn litter.

According to the clinical observations, increased expression of the neurotrophic factors provides protection for a neonate even if hypoxia factors are present and realized. A low content of BDNF and GDNF was observed in the group of infants with a high risk of developing unfavorable hypoxic damaging effects.

The protective role of BDNF and GDNF in the regulation of homeostasis in the fetuses exposed to chronic hypoxia has been established experimentally and clinically.
